# ARGLU1 is a negative regulator of the adenoviral replicative cycle

**DOI:** 10.1128/jvi.01407-25

**Published:** 2025-11-04

**Authors:** Amit Koul, Lauren Fulham, Nikolas Akkerman, Drayson Graves, Esha Kaul, Khadija Khadija, Peter Pelka

**Affiliations:** 1 Department of Microbiology, University of Manitoba 468335, Winnipeg, Manitoba, Canada; 2 Department of Medical Microbiology and Infectious Diseases, University of Manitoba 574854, Winnipeg, Manitoba, Canada; University of Toronto, Toronto, Ontario, Canada

**Keywords:** ARGLU1, human adenovirus, E1A, transcription, DNA damage response

## Abstract

We have previously reported that human adenovirus E1A interacts with ARGLU1, a small disordered cellular protein. The consequences of this interaction for virus replication were unclear. E1A is the first protein produced during adenovirus infection. Via protein-protein interactions, E1A modifies the cellular environment to create optimal conditions for viral replication. To better understand the molecular mechanisms driving viral infection, we further investigated the functional consequences of the interaction between ARGLU1 and E1A. ARGLU1 interacts with E1A directly as determined by a GST-pulldown assay with recombinant proteins. Importantly, ARGLU1 was found to act as a transcriptional repressor when it was localized to viral promoters. Repression was driven by enhanced promoter-proximal RNA polymerase II pausing. Significantly, ARGLU1-induced repression of viral promoters is likely an unintended consequence of ARGLU1 binding to E1A, as a mutant of E1A (*dl*1102), unable to bind to ARGLU1, did not show reduced viral gene expression. Furthermore, ARGLU1 was found to colocalize with E1A in infected cell nuclei. Finally, the binding of E1A to ARGLU1 appears to reduce DNA damage repair in bleomycin-treated and adenovirus-infected cells, suggesting that E1A binding to ARGLU1 is important for DNA damage response inhibition. Overall, this study underscores the complexity of virus-host interactions and reveals a novel role for ARGLU1 during adenoviral infection.

**IMPORTANCE:**

This study uncovers a novel antiviral role for ARGLU1, known for its involvement in splicing, transcriptional regulation, and DNA damage repair. Our study demonstrates that ARGLU1 functions as a viral restriction factor by reducing virus growth through the inhibition of viral gene expression and enhanced DNA damage response, leading to reduced viral growth. These findings not only highlight an important role for ARGLU1 in host antiviral mechanisms but also emphasize the utility of adenovirus as a tool to uncover critical cellular pathways.

## INTRODUCTION

Arginine and glutamate-rich protein 1 (ARGLU1) is a small intrinsically disordered protein (IDP) involved in regulating transcription, RNA processing, and DNA damage repair (1–3). Despite its disordered nature, ARGLU1 is evolutionarily conserved and features two distinctly charged regions—a positively charged arginine-rich N-terminus and a negatively charged C-terminus. These regions enable interactions with the spliceosome and nuclear receptors, respectively (2, 4). Initially, ARGLU1 was identified as a co-regulator of estrogen receptor-mediated transcription through its interaction with mediator subunit 1 (MED1), promoting breast cancer cell growth (1). Subsequent research revealed its role as a modulator of glucocorticoid signaling in the central nervous system, where its deletion significantly alters gene expression and alternative splicing linked to neurogenesis (2). Moreover, loss of ARGLU1 function has been associated with global splicing defects and neuronal deficiencies (5). Recent findings have extended the role of ARGLU1 in viral gene regulation and DNA damage repair (3). Knockdown of ARGLU1 enhances viral gene expression and growth, whereas its presence supports the DNA damage repair response (3). Mechanistically, ARGLU1 impedes transcription elongation by promoting promoter-proximal pausing of RNA polymerase II (RPII). This occurs through its binding to Jumonji-domain-containing protein 6 (JMJD6), which displaces Bromodomain-containing protein 4 (BRD4), a key partner required for transcriptional elongation (3). By stabilizing RPII pausing, ARGLU1 enhances resistance to genotoxic drugs and facilitates DNA repair processes (3). RNA polymerase II pausing is a regulatory step in gene regulation and productive RNA synthesis (6). DNA lesions in the template strand cause stalling of RNA polymerase II, leading to genome-wide transcription arrest until the damage is mended via transcription-coupled repair mechanisms (7). Together, these findings suggest that ARGLU1 plays a diverse role in multiple cellular processes, a common feature of IDPs in cells (8).

Human adenovirus (HAdV) is a small, nonenveloped DNA virus with a double-stranded linear genome that primarily infects terminally differentiated epithelial cells (9). As the first viral protein expressed during infection, the immediate early gene product Early protein 1A (E1A) plays a central role in activating the viral transcriptional program and reprogramming the host cell environment to facilitate viral replication (10, 11). E1A achieves this by interacting with a wide array of cellular proteins, including cell cycle regulators, transcription factors, chromatin remodelers, and transcriptional coregulators, thereby disrupting normal cellular homeostasis and driving viral gene expression (10, 11). In addition to its pivotal role in viral replication, E1A has been effectively employed as a molecular probe to uncover key regulators of cellular pathways, many of which are implicated in cancer biology, for example, FUBP1, Nek9, DREF, Ku70, and RuvBL1 (12–16). Notably, E1A was recently found to bind ARGLU1 in HAdV-B7-infected cells through the N-terminus of E1A, with unknown consequences for the adenoviral replicative cycle (3). Nevertheless, these findings add to the growing understanding of how E1A manipulates host cell machinery and highlight the broader utility of HAdV in dissecting complex cellular regulatory networks (9, 17, 18).

Adenoviruses have evolved multiple strategies to subvert the host DNA damage repair (DDR) response, a key genome integrity-maintaining mechanism that is deleterious to the replication of many viruses (19). The presence of viral genomes will trigger DDR in the infected cells; to overcome this, HAdVs have adapted to evade or inhibit these responses to ensure efficient replication. Interference with the activities of the Mre11–Rad50–Nbs1 (MRN) complex—a central sensor consisting of three subunits, Mre11, Rad50, and Nbs-1—is crucial for efficient viral replication (20–22). Additionally, adenoviral protein E1B-55K, together with E4orf6, promotes the degradation of DDR components, including Tip60 (23), an ATM activator, thereby suppressing DDR signaling. Structurally, adenoviral genomes and replicating viral genomes resemble DNA double-stranded breaks (DSBs) due to their linear nature, but the presence of a covalently attached terminal protein (TP) at the 5’ ends protects them from recognition by DNA break sensors (19). Similarly, replication intermediates containing extensive single-stranded DNA could potentially trigger ATR signaling; however, they are shielded by the adenoviral DNA-binding protein (DBP) (19). Moreover, wild-type HAdV genomes do not undergo concatemerization (a process commonly associated with unprotected DNA ends), suggesting that adenoviral proteins actively suppress the DDR machinery (19, 24). Despite these suppression strategies, the DDR can still be inadvertently activated. For example, the covalent linkage of TP to the viral DNA may be recognized as a DNA–protein crosslink (DPC), triggering DPC repair pathways (21), exposing the adenoviral genome and activation of DDR. This can lead to TP removal and consequently impair viral genome integrity and replication efficiency.

In the present study, we investigated the functional significance of the interaction between ARGLU1 and E1A. We demonstrate that E1A and ARGLU1 interact directly via a GST-pulldown assay with bacterially expressed proteins. Overexpression of ARGLU1 resulted in a significant reduction of viral gene expression in cells infected with the phenotypically wild-type HAdV5 *dl*309, but not in cells infected with the HAdV5 mutant *dl*1102, which lacks the ability to bind ARGLU1. Consistently, ARGLU1 overexpression also led to significant changes in adenoviral protein expression. Although viral genome copy numbers were not significantly different between *dl*309 and *dl*1102-infected HT1080 (HT) and HT1080 cells overexpressing ARGLU1 (HT-A) cells, ARGLU1 overexpression substantially impaired viral growth. Additionally, chromatin immunoprecipitation (ChIP) further demonstrated that ARGLU1 is recruited to adenoviral promoters in *dl*309-infected HT-A cells. Importantly, comet assays revealed reduced DNA damage repair in *dl*309-infected cells, suggesting that disruption of DNA damage repair-related ARGLU1 activities is important for viral replication.

## MATERIALS AND METHODS

### Antibodies

Mouse monoclonal anti-E1A M2, M37, M58, and M73 antibodies were previously described (25) and were grown in-house and used as the hybridoma supernatant. For immunoprecipitations (IPs), 25 µL was used, and for western blot assays, a dilution of 1:400 was used. 12CA5 anti-hemagglutinin (anti-HA) mouse monoclonal antibody was grown in-house and used as hybridoma supernatant; 25 µL of hybridoma supernatant was used in chromatin immunoprecipitation (ChIP) experiments. Mouse monoclonal anti-72k DNA-binding protein (DBP) antibody was previously described (26) and was used at a dilution of 1:400 for western blotting. Viral structural and late proteins were detected with anti-adenovirus type five antibody from Abcam (cat. # ab6982) at a dilution of 1:5,000. Rat monoclonal anti-hemagglutinin (anti-HA) clone 3F10 was obtained from MilliporeSigma (cat. #11867423001). Anti-RNA polymerase II (CTD) was obtained from Abcam (cat. # ab252854). Antibody for human ARGLU1 was purchased from Invitrogen (cat. # PA5-66041); additionally, antibody for human ARGLU1 was also generated by Pacific Immunology using the following peptide sequence: Cys-KEEQKIILGKGKSRPKLSFSLKTQD. The antibody was affinity-purified using peptide columns before use. Antibodies for ATM (cat. # 2873), phospho-serine 1981-ATM (cat. # 13050), ATR (cat. # 13934), phospho-threonine 1989-ATR (cat. # 30632), Chk1 (cat. # 2360), phospho-serine 345-Chk1 (cat. # 2348), p53 (cat. # 9282), and p21 (cat. # 2947) were purchased from Cell Signaling Technologies. Anti-53BP1 antibody (cat. # ab175933) was purchased from Abcam. Secondary antibodies were purchased from Jackson ImmunoResearch.

### Cell and virus culture

HT1080 cells (ATCC# CCL-121) were cultured in Dulbecco’s modified Eagle’s medium (MilliporeSigma) supplemented with 5% fetal bovine serum, streptomycin, and penicillin (Corning). Unless stated otherwise, all viral infections were performed at a multiplicity of infection (MOI) of 10 in serum-free medium for 1 h, after which fresh complete medium was added without removing the infection medium. HT1080 cells overexpressing ARGLU1 (HT-A) were generated as previously described (3). HAdV5 *dl*309 was previously described (27), as was *dl*1102 mutant (28).

### Protein purification and GST pulldown assay

Human ARGLU1 was expressed as a GST-tagged fusion protein (GST-ARGLU1) in *E. coli* BL21 (DE3)-CodonPlus RIPL (Agilent Technologies). Cells were lysed on an EmulsiFlex C3 (Avestin) in Buffer-A (20 mM HEPES (pH 7.4), 300 mM NaCl, 0.1 mM EDTA, 10% (vol/vol) glycerol, 2 mM DTT, and 1% Triton X-100). Crude lysates were clarified by centrifugation at 35,000 × *g* for 30 min, at 4°C. Clarified lysates were affinity-purified using glutathione Sepharose (Thermo Fisher Scientific), followed by elution with 40 mM reduced glutathione. The eluted protein was dialyzed in Buffer-B (20 mM HEPES (pH 7.4), 100 mM NaCl, 0.1 mM EDTA, 5% (vol/vol) glycerol, 2 mM DTT) overnight at 4°C. Protein was concentrated to 1 mg/mL, concentration was determined via Bradford assay, and protein was stored at 4°C. HAdV5 E1A289R was expressed as a 6xHis-tagged fusion protein (6xHis-E1A289R) in BL21 (DE3)-CodonPlus RIPL (Agilent Technologies) and purified with an Ni-NTA affinity column as described previously (26, 29). Recombinant 6xHis-E1A289R was concentrated to 1 mg/mL, concentration was determined via Bradford assay, and protein was stored at −20°C. GST pulldowns were performed as described previously (29, 30).

### Western blot

Protein samples were boiled in sample buffer containing 100 mM DTT at 95°C for 5 min. Samples were resolved on a Bis-Tris Bolt Plus 4%–12% protein gel (Invitrogen) using either MOPS or MES buffer (Invitrogen), depending on the size of the target protein. Following electrophoresis, proteins were transferred to a PVDF membrane using the eBlot L1 blot transfer apparatus (Genscript) using the default protocol. Membranes were then blocked for 1 h in 5% skim milk powder dissolved in TBST. Primary antibodies were applied in 1% BSA or 5% skim milk powder in TBST, shaking, overnight at 4°C. Horseradish peroxidase (HRP)-conjugated secondary antibodies (Jackson ImmunoResearch) were applied in blocking buffer at a 1:100,000 dilution. Protein bands were visualized using the Azure C600 digital imager (Azure Biosystems) with Luminata Forte ECL reagent (MilliporeSigma).

For the phosphoprotein blot, the lysis buffer used was the NP-40 lysis buffer supplemented with 10 mM sodium phosphate, 10 mM sodium orthovanadate, and 10 mM beta-glycerophosphate; protein samples were resolved on a Bis-Tris Bolt Plus 4%–12% protein gel (Invitrogen) using MOPS (Invitrogen); and a western blot was performed as above.

### Chromatin immunoprecipitation

The formaldehyde cross-linking and chromatin immunoprecipitation (ChIP) were carried out as described previously (3, 31). Cells were infected with HAdV5 strain *dl*309 or *dl*1102 at an MOI of 50 and harvested 24 h after infection for ChIP analysis. Mock-infected cells were harvested at the same time. For immunoprecipitation of E1A, a combination of monoclonal M73 and M58 antibodies was used. Rabbit anti-rat antibody was used as a negative IgG control (MilliporeSigma). qPCR reactions were carried out using Powerup SYBR Green Master Mix (Thermo Fisher Scientific) on 2.6% of total eluted ChIP DNA as template, by BioRad CFX96 Real Time PCR instrument (Bio-Rad) according to manufacturer’s instructions. The annealing temperature used was 69°C, and 40 cycles were run. Primers for viral and cellular promoters are provided in Table 1.

**TABLE 1:** PCR primers used in this study

Gene	Forward sequence	Reverse sequence
GAPDH	GAGTCAACGGATTTGGTCGT	TTGATTTTGGAGGGATCTCG
E1A	CACGGTTGCAGGTCTTGTCATTAT	GCTCAGGTTCAGACACAGGACTGTA
E1B	CGCGCTGAGTTTGGCTCTAG	TCAAACGAGTTGGTGCTCATG
E2A	GGGGGTGGTTTCGCGCTGCTCC	GCGGATGAGGCGGCGTATCGAG
E3A	GCCGCCACAAGTGCTTTG	CTCGGAGAGGTTCTCTCGTAGACT
E4orf3	CTCGAGTTATTCCAAAAGATTATCCAAAACC	GAATTCATTCGCTGCTTGAGGCTGAA
FIBER	ATGCTTGCGCTCAAAATGGG	TTTTTGAGAGGTGGGCTCAC
DBP	TTAAGCCCCAGCCAATCATG	TGCCTCGTTGTTCTTGTACC
HEXON	CTTACCCCCAACGAGTTTGA	GGAGTACATGCGGTC CTTGT
E3p for ChIP	CGCGGGACCCCACATGATAT	CGCCCTCTGA TTTTCAGGTG
E4p for ChIP	TAAACACCTGAAAAACCCTCCTGCC	GGCTTTCGTTTCTGGGCGTA
E2p for ChIP	AGCAAATACTGCGCGCTGAC	AGAATTCGGTTTCGGTGGGC
MLPp for ChIP	TCGGCCTCCGAACGGTAAGA	AACTTTATGCCTCGCGCGGG

### Immunofluorescence

HT or HT-A cells were plated at low density (∼40,000 cells per chamber) on Nunc Lab Tek-II chamber slides (Thermo Fisher Scientific) and subsequently infected as described above. Twenty-four hours after infection, the cells were fixed in 4% formaldehyde, permeabilized using 0.1% Triton X-100, and blocked in blocking buffer (1% normal goat serum, 1% BSA, 0.2% Tween-20 in PBS), and stained with specific primary antibodies. M73 was used neat (hybridoma supernatant), E2 DBP antibody was used at a 1:50 dilution (hybridoma supernatant), and ARGLU1 antibody was used at a dilution of 1:100. Alexa Fluor 488 and 594-conjugated secondary antibodies (Jackson ImmunoResearch) were applied at a 1:600 dilution. Following three 10 min washes with PBS-T, slides were mounted using Prolong Gold with DAPI (4’,6-diamidino-2-phenylindole) (Invitrogen) and imaged using Zeiss LSM700 confocal laser scanning microscope. Images were analyzed using the Zeiss ZEN software package version 8.

### Real-time gene expression analysis

HT and HT-A cells were cultured in a 6-well plate (~1 million cells per well) and infected with *dl*309 and *dl*1102 at an MOI of 10. Total RNA was extracted at the indicated time points using TRIzol Reagent (MilliporeSigma), following the manufacturer’s instructions. To eliminate contaminating DNA, the total extracted RNA was treated with the TURBO DNA-free kit (Invitrogen) according to the manufacturer’s protocol. RNA concentration was measured using A260, and its quality was assessed based on the A260/A280 ratio. For cDNA synthesis, 1 µg of total RNA was reverse-transcribed using the SuperScript VILO reverse transcriptase master mix (Invitrogen) with random hexanucleotide primers, following the manufacturer’s protocol. Next, 2% of the total cDNA was then used for real-time expression analysis on a BioRad CFX96 real-time thermocycler (Bio-Rad) using BioRad Sso Advanced Universal SYBR Green Supermix in a 10 µL reaction volume, with the standard cycling program recommended for this reagent. Data acquisition was performed using BioRad CFX Manager software (version 3.1). Each sample was analyzed with at least three biological and two technical replicates, unless stated otherwise in the figure legend. Analysis of expression data was carried out using the percentage of glyceraldehyde 3-phosphate dehydrogenase (GAPDH) method, with raw Cq values for GAPDH serving as an internal quality control, as its expression remained stable across experimental conditions. The specificity of PCR amplicons was confirmed via melt curve analysis. To verify the absence of contaminating DNA, RNA samples were also subjected to qPCR analysis without reverse transcription. Post-acquisition data analysis was conducted using Excel for Office 365 for Windows.

### Virus growth assay

HT and HT-A cells were infected with HAdV5 *dl*309 at an MOI of 10. The virus was allowed to adsorb for 1 h at 37°C under 5% CO₂. Following adsorption, the infection media were topped off with fresh complete media. The cells were then incubated at 37°C under 5% CO₂ for the remainder of the experiment. Each time point was done in three independent biological replicates. Viral titers were measured at 48 and 72 h after infection by plaque assays on 293 cells; titers for each biological replicate were determined in triplicate, and the average of these technical replicates was used in the final calculation. Ratios of virus growth in HT-A versus HT cells were calculated by dividing the viral titer obtained in HT-A cells with each virus at a given time point by the viral titer for the same virus and time point obtained in HT cells. Ratio values of less than 1 represent reduced growth in HT-A cells compared with HT, values greater than 1 represent enhanced growth in HT-A cells versus HT, and values equal to or near 1 indicate similar growth in HT-A cells compared with HT.

### Comet assay

HT and HT-A cells (~100,000 cells per well) were infected with HAdV5 *dl*309 or *dl*1102 at an MOI of 500 for 24 h. Following infection, the cells were treated with 5 µM bleomycin (Selleck Chemical) for 15 min, then allowed to recover for 0–30 min. After the recovery period, DNA damage assessment was performed using the Comet Assay Kit (Abcam, cat. ab238544) according to the manufacturer’s protocol. Comet tails were resolved through alkaline electrophoresis, stained, and imaged using the ImageXpress Micro 4 Imager (Molecular Devices) with MetaXpress software (version 6.7.0.211). Image analysis and quantification of comet parameters were conducted using ImageJ software with the OpenComet plug-in (32) or TriTek CometScore 2.0.

### Statistical analysis

All statistical analyses were conducted using GraphPad Prism v5 software, applying a two-tailed Student’s *t*-test. A *P*-value of ≤0.05 was considered statistically significant. Data are presented as mean values, with error bars representing the standard deviation of all biological and technical replicates. The number of biological replicates (n) is specified in the corresponding figure legends.

### PCR primers

Table 1 lists the PCR primers used in quantitative PCR analysis, viral gene expression analysis, and ChIP of viral genes. Small letter p at the end of the gene name denotes promoter primers used for ChIP.

## RESULTS

### ARGLU1 directly interacts with E1A and inhibits virus growth

**Fig 1:**
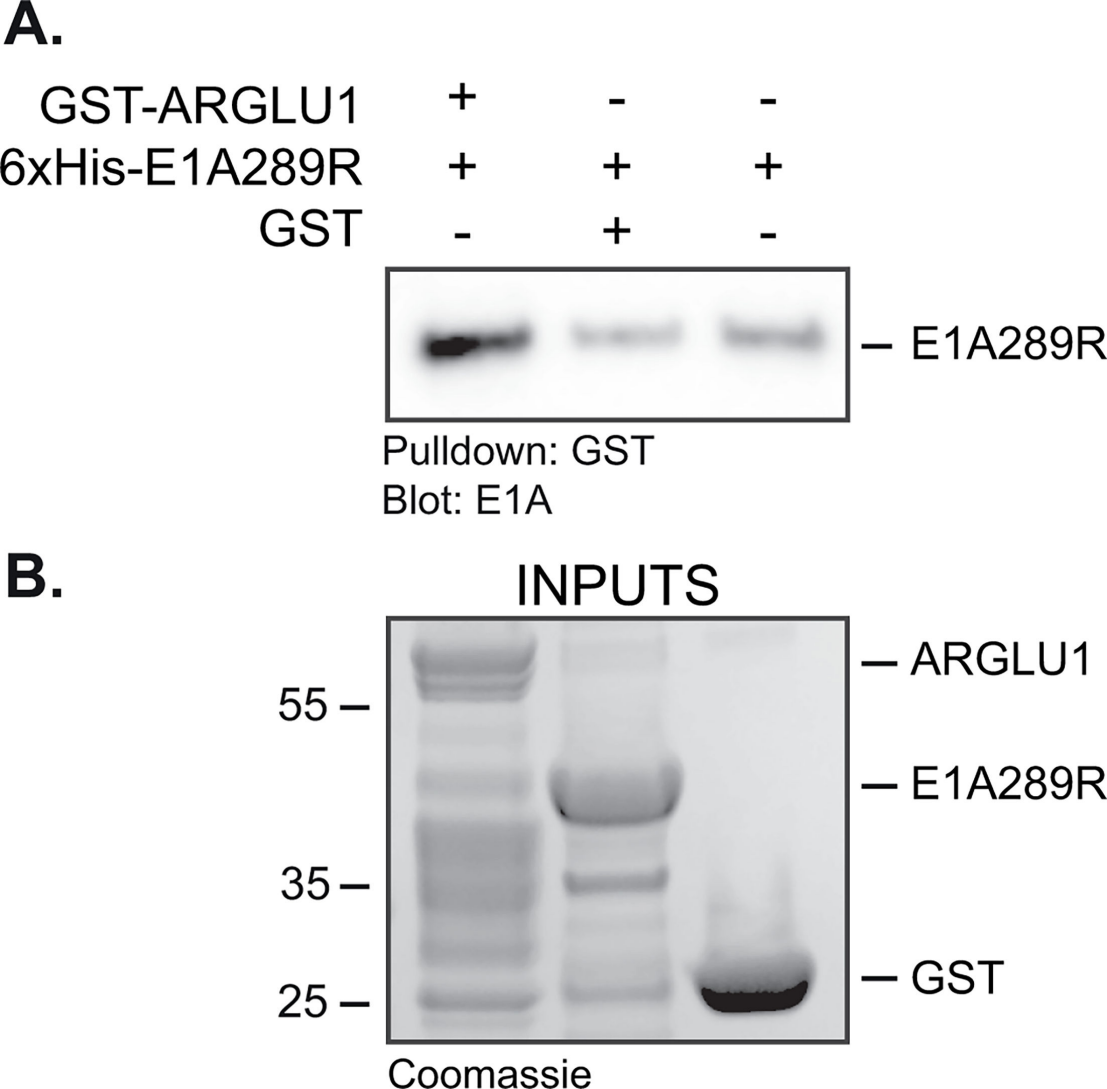
E1A directly interacts with ARGLU1 *in vitro*. (**A**) *E. coli*-expressed 6xHis-E1A was mixed with GST-ARGLU1 or GST, also expressed in *E. coli*, and a GST pull-down was performed using glutathione beads, followed by western blotting with M73 and M2 monoclonal E1A antibodies. Glutathione beads alone were used as a negative control; 1 µg of each protein was used in the pull-down assay. (**B**) SDS polyacrylamide gel of input GST-ARGLU1, 6xHis-E1A289R, and GST used in the pulldown assay stained with Coomassie Brilliant Blue.

Mass spectrometry analysis of protein complexes associated with HAdV-B7 E1A has previously identified ARGLU1 as a binding partner that interacts with the N-terminus of E1A (3). To determine whether this was a direct interaction, we performed a GST-pulldown assay with bacterially expressed 6xHis-E1A289R and GST-ARGLU1 (Fig. 1). Although 6xHis-E1A289R was efficiently pulled down by GST-ARGLU1, GST alone did not pull down significant amounts of 6xHis-E1A289R (Fig. 1). These results demonstrate that E1A directly interacts with ARGLU1.

**Fig 2:**
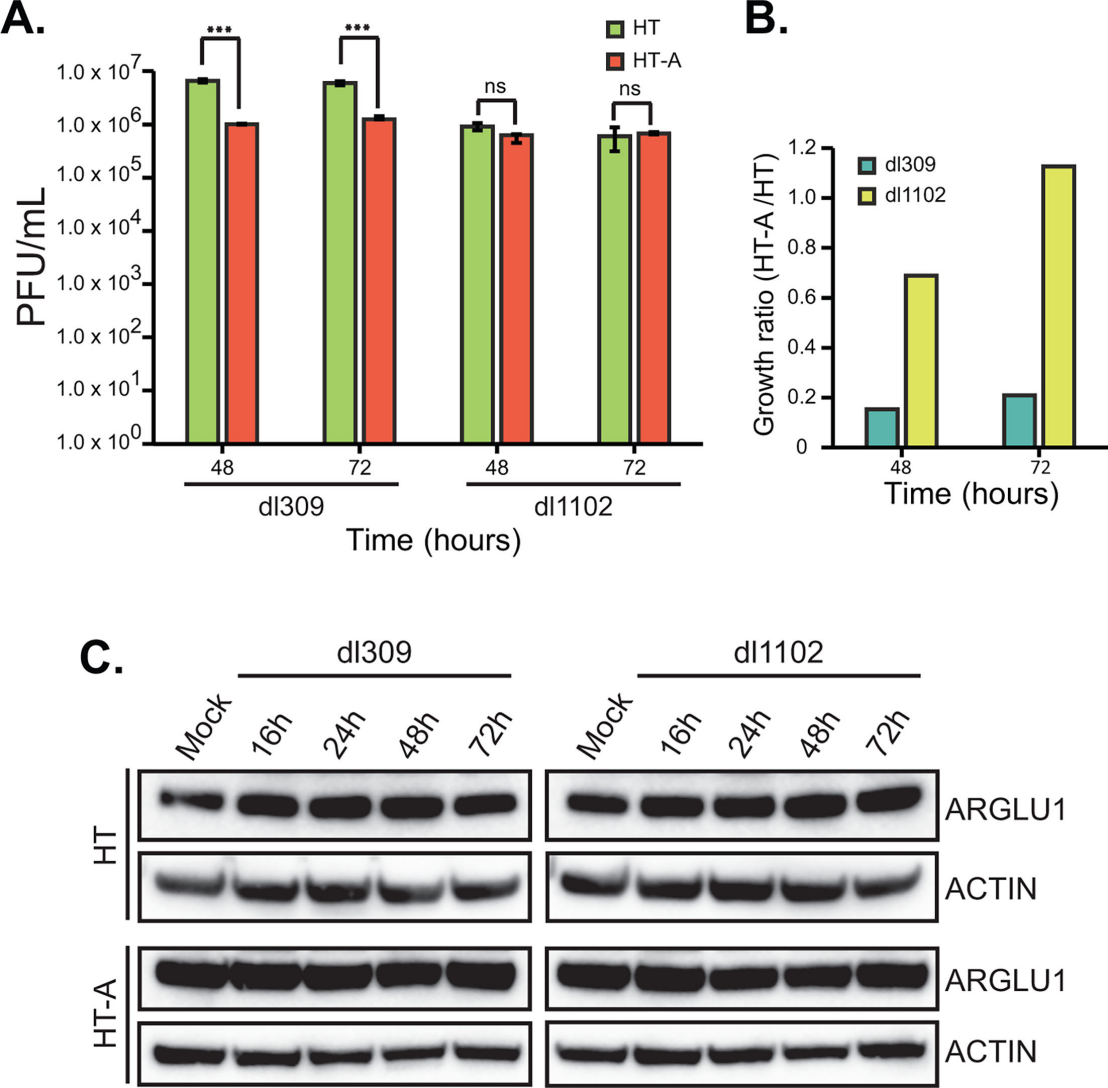
Overexpression of ARGLU1 limits HAdV5 growth. (**A**) HT and HT-A cells were infected with HAdV5 *dl*309 or *dl*1102 at an MOI of 10. The virus was harvested, and the titer was determined on 293 cells at the indicated time points after infection of HT or HT-A cells. Error bars represent standard deviation (*n* = 3); ***P ≤ 0.001. (**B**) Ratios of HT-A virus growth over HT from panel **A**. (**C**) Western blots of ARGLU1 and ACTIN from HT and HT-A cells infected with *dl*309 or *dl*1102 at an MOI of 10 for the indicated time. For these blots, 20 µg of total protein was resolved on 4-12% gradient NuPAGE BOLT gels and blotted for the indicated proteins. ns, not significant.

To explore the role of ARGLU1 during adenovirus infection, we examined its impact on viral replication using phenotypically wild-type HAdV5 *dl*309 in HT and HT-A cells and similarly a deletion mutant, *dl*1102, that expresses E1A deficient for ARGLU1 binding (3). HT-A cells displayed significantly reduced *dl*309 growth, with approximately 8-fold reduction in virus titers at 48 and 72 h after infection compared with HT cells (Fig. 2). The reduction in viral titers observed in HT-A cells suggests that ARGLU1 may act as a viral restriction factor, impeding adenoviral growth or assembly, without impacting viral genome replication (data not shown). Conversely, *dl*1102 was minimally, and not significantly, affected in growth by ARGLU1 overexpression at 48 and 72 h after infection (Fig. 2). Finally, we determined whether infection affects endogenous and overexpressed ARGLU1 protein levels (Fig. 2); no significant difference was observed in ARGLU1 levels early in infection, with a minimal reduction late in infection. Collectively, these findings indicate that ARGLU1 directly interacts with E1A and plays a negative role in HAdV5 replication.

### ARGLU1 affects viral gene and protein expression

**Fig 3:**
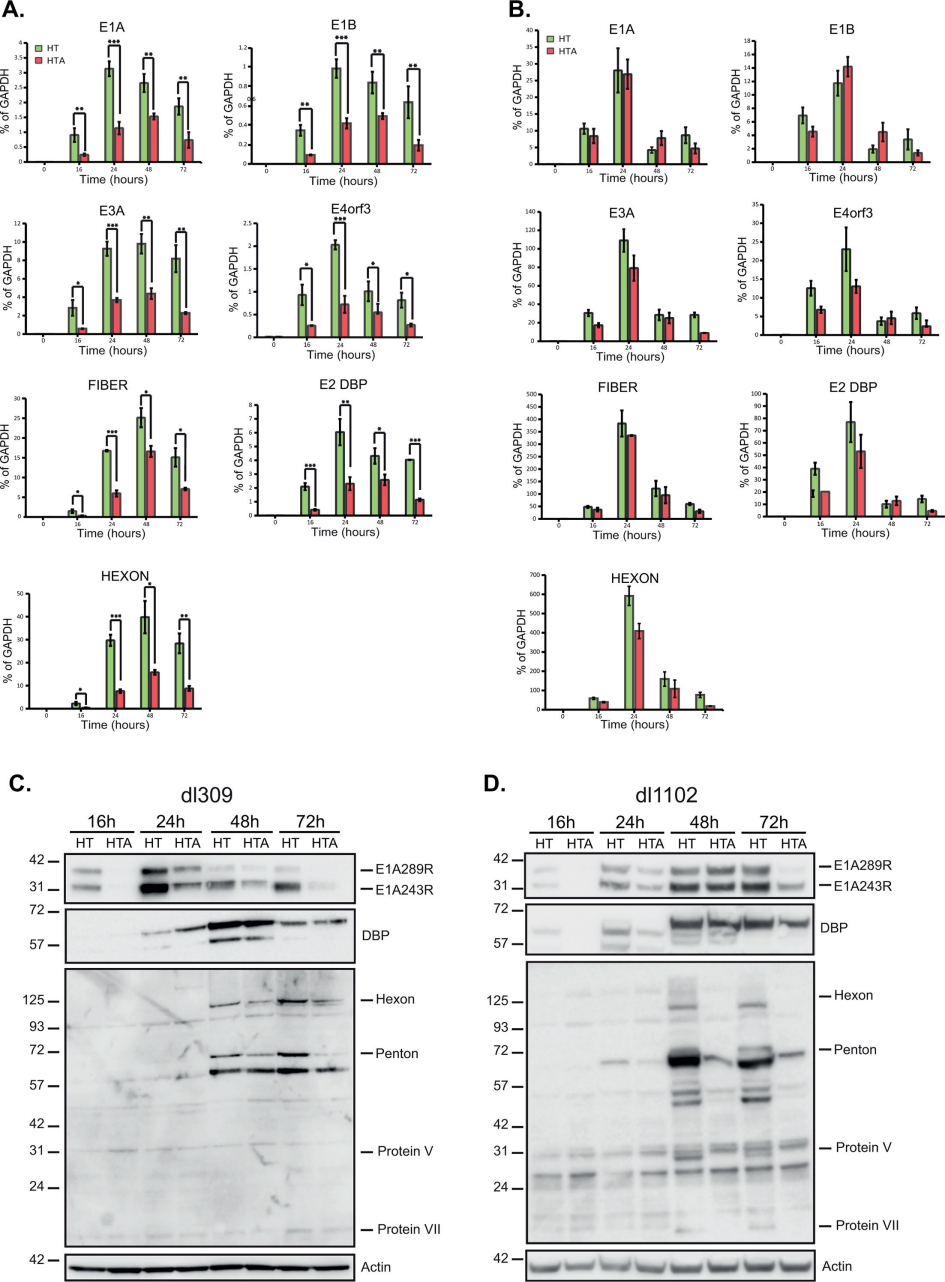
ARGLU1 alters HAdV5 gene and protein expression. (**A**) HT and HT-A cells were infected with HAdV5 *dl*309 at an MOI of 10; the cells were then harvested at the indicated time points, and total RNA was converted to cDNA using VILO Reverse Transcriptase Master Mix. Expression levels of viral genes—*E1A*, *E1B*, *E3A*, *E4orf3*, *fiber*, *DBP,* and *hexon*—were determined by qPCR at the indicated time points. Error bars represent standard deviations (*n* = 3) *P ≤ 0.05, **P ≤ 0.01, ***P ≤ 0.001. (**B**) Same as (**A**) except HT and HT-A cells were infected with HAdV5 *dl*1102. Error bars represent standard deviations (*n* = 3); none of the differences were statistically significant. For all qPCR analyses, time zero represents uninfected cells and *GAPDH* mRNA was used as the normalization control. (**C**) HT and HT-A cells were infected with HAdV5 *dl*309 at an MOI of 10, at the indicated times after infection cells were lysed in NP-40 lysis buffer, and 20 µg of total protein was loaded per well into a 4-12% NuPAGE BOLT polyacrylamide gel and blotted for the indicated proteins. ACTIN was used as a loading control. (**D**) Same as C except that HAdV *dl*1102 was used in the infection.

The reduced viral growth observed in HT-A cells (Fig. 2) and enhanced growth when ARGLU1 is knocked down (3) suggest that ARGLU1 may either directly interfere with viral replication or contribute to host innate antiviral response. We therefore wanted to investigate whether overexpression of ARGL1 affects viral gene expression, as our earlier studies with ARGLU1 knockdown showed enhanced viral gene expression with reduced ARGLU1 levels (3). To elucidate this, HT and HT-A cells were infected with HAdV5 *dl*309 or *dl*1102, and total RNA was extracted, and expression of viral genes was analyzed at 16, 24, 48, and 72 h after infection. Most viral genes are negatively affected by ARGLU1 overexpression in *dl*309-infected cells (Fig. 3). In line with our observation of reduced viral titers in HT-A, compared with HT cells, we observed a significant overall reduction in viral gene expression in HT-A cells infected with *dl*309 (Fig. 3). At 16 h, DBP mRNA in HT-A showed the largest reduction in gene expression, followed by *E1A* and *E1B* compared with HT cells (Fig. 3A). Expression of viral genes, *E1A*, *E1B*, *E3A*, *E4orf3*, *fiber,* and *hexon,* was also significantly reduced at 24 h in HT-A compared with HT cells (Fig. 3A). Additionally, significant reduction in gene expression was observed at 48 h for *E1A*, *E1B,* and *E3A* (Fig. 3). However, mRNA levels of E4orf3, fiber, DBP, and hexon showed a modest reduction at 48 h. Furthermore, at 72 h, with the exception of mRNAs for E4orf3 and fiber, all viral genes showed significant reduction in gene expression in HT-A in comparison to HT cells. Our observation that ARGLU1 overexpression in HT-A cells led to decreased levels of E3A and E4orf3 mRNAs, compared with HT cells, suggests that ARGLU1 may downregulate some elements of viral gene expression. The observed results are unlikely to be attributed to variability in the normalization control (*GAPDH*), as the raw quantification cycle (Cq) values remained consistent across samples. Importantly, HAdV5 mutant *dl*1102 that deletes E1A residues 26–35 (28, 33) required for ARGLU1 binding by E1A (3) was assayed for viral gene expression in HT and HT-A cells. With this mutant, we did not observe any significant reduction in viral gene expression in HT-A compared with HT cells (Fig. 3).

To determine whether altered levels of mRNA translate to protein expression, we performed western blotting for E1A, DBP, and the late proteins in HT and HT-A cells at 16, 24, 48, and 72 h after infection with either *dl*309 or *dl*1102 (Fig. 3). We observed a reduction in levels of E1A proteins in HT-A compared with HT cells with both viruses. A major reduction in E1A protein expression was observed very early in the infection at 16 h, whereas E1A showed a modest reduction at 48 h after infection, consistent with the mRNA levels. The observed decrease in E1A levels at 16 h after infection for HT-A cells correlates with decreased mRNA expression, suggesting that the decline may be transcriptionally driven. Levels of DBP were broadly similar between HT and HT-A cells with minimal differences throughout infection, except at 24 h in *dl*309-infected cells, where slightly higher levels were observed in HT-A cells compared with HT, whereas the opposite was observed in *dl*1102-infected cells. We have also observed a marked decrease in protein levels for hexon and penton at 48 and 72 h after infection (Fig. 3). The reduced protein levels for hexon consistently correlated with mRNA levels at both 48 and 72 h time points. One major difference observed between *dl*309 and *dl*1102 was that overall protein levels were considerably higher in the latter, consistent with higher gene expression. Protein levels of E1A and DBP sustained higher expression late in infection, whereas *dl*309-infected samples showed greater reduction late in infection. Together, these results demonstrate that ARGLU1 is a negative regulator of viral gene and protein expression during infection.

### ARGLU1 sub-cellular distribution is altered during infection

**Fig 4:**
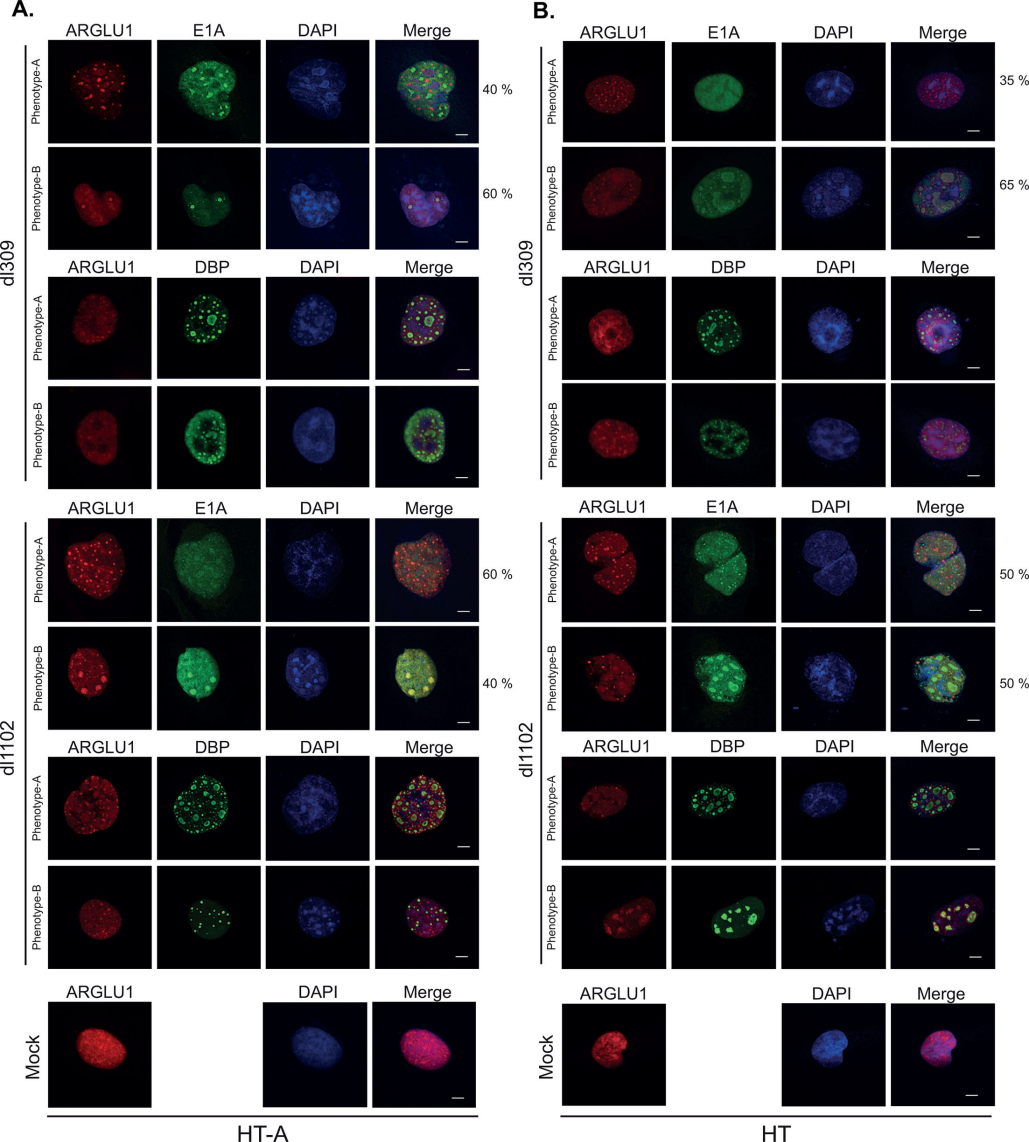
ARGLU1 sub-nuclear localization is altered in infected cells. (**A**) HT-A cells were infected with HAdV5 *dl*309 or *dl*1102 at an MOI of 10. Infected cells were stained for ARGLU1, E1A, and DBP using anti-ARGLU1, anti-E1A (M73), and anti-DBP antibodies 24 h after infection. Alexa 488 and Alexa 594-conjugated secondary antibodies were used to visualize ARGLU1, E1A, and DBP. DAPI was used as a nuclear counterstain. Images were acquired with a Zeiss LSM700 confocal laser scanning microscope using a 63× objective lens. Representative images of the observed phenotypes are shown. (**B**) Same as (**A**) except HT cells were infected with HAdV5 *dl*309 or *dl*1102 at an MOI of 10. Percentages represent the observed frequency of the shown ARGLU1 phenotype. Bar represents 5 µm.

Since ARGLU1 influences viral replication and gene expression, we examined its subcellular localization in infected HT and HT-A cells. In uninfected cells, ARGLU1 showed predominantly a diffuse nuclear localization with some areas of concentration or speckling (Fig. 4), which is consistent with our previous observations (3). In infected cells, two distinct ARGLU1 phenotypes (phenotype-A & phenotype-B) were observed when cells were stained for ARGLU1 and E1A or DBP, the latter to label viral replication compartments (Fig. 4). ARGLU1 was found either as a diffuse nuclear protein with no specific foci, similar to what was observed in uninfected cells (Fig. 4) or it was re-localized to distinct spots within the nucleus, some of which co-localized with viral replication centers (Fig. 4). The re-localization of ARGLU1 within the infected cells was not dependent on the ability of ARGLU1 to bind to E1A as the mutant *dl*1102 showed similar ARGLU1 phenotype to *dl*309, suggesting that it is infection or virus replication that is causing alteration in sub-nuclear ARGLU1 distribution rather than E1A binding *per se*. Overall, ARGLU1 phenotypes in infected and uninfected HT cells (Fig. 4) were similar to those observed in the ARGLU1-overexpressing HT-A cells. Variability in E1A distribution was also observed, but this is consistent with our previous observations (14, 30) and not a direct consequence of ARGLU1 overexpression. Collectively, these results demonstrate that during viral infection, the sub-nuclear distribution of ARGLU1 is altered.

### ARGLU1 is recruited to viral promoters during HAdV infection

**Fig 5:**
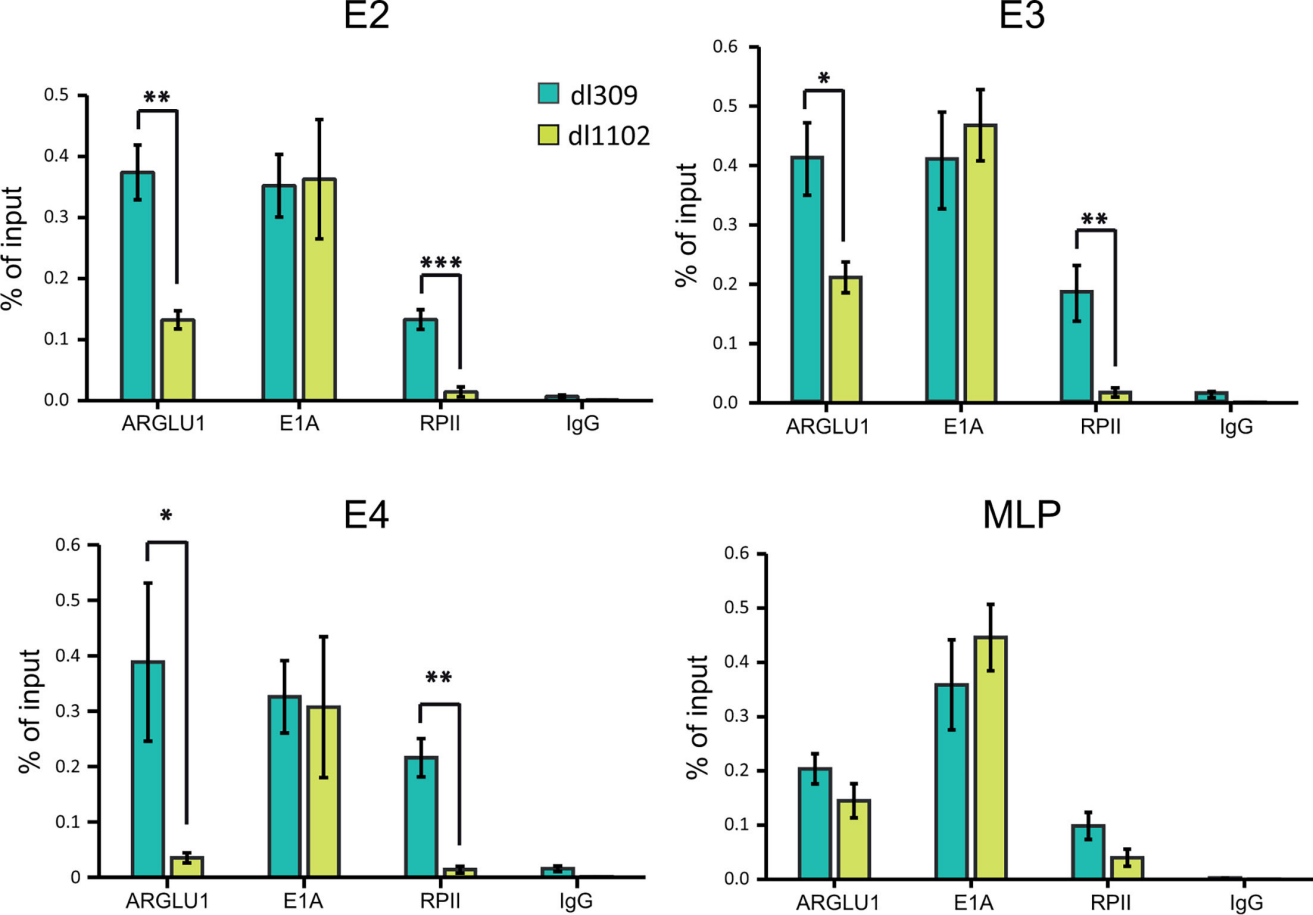
ARGLU1 is recruited to viral promoters in an E1A-dependent manner. HT-A cells were infected with HAdV5 *dl*309 or *dl*1102 at an MOI of 50; 24 h after infection, the cells were fixed, and chromatin was immunoprecipitated as described in Materials and Methods, using a cocktail of M73 and M58 antibodies for E1A, rabbit anti-ARGLU1 antibody, rat anti-RPII antibody, or rabbit anti-rat IgG antibody as the IgG control. Following immunoprecipitation and DNA purification, the samples were analyzed with a BioRad CFX96 real-time PCR instrument and plotted as a percentage of the input. Error bars represent standard deviation (*n* = 3); *P ≤ 0.05, **P ≤ 0.01, ***P ≤ 0.001.

The observed changes in viral gene expression upon ARGLU1 overexpression in HT-A cells suggested that ARGLU1 may play a role in transcriptional regulation. This is consistent with previous findings showing that ARGLU1 knockdown enhances viral gene expression (3), whereas our data suggested that overexpression of ARGLU1 significantly reduced viral gene expression (Fig. 3). Based on these findings, we decided to investigate if the association of ARGLU1 with E1A may enable it to associate with viral promoter regions. To determine whether ARGLU1 associates with viral promoters, and what effect this has on the presence of RPII proximal to these promoters, we performed ChIP assay on *dl*309- and *dl*1102-infected HT-A cells (Fig. 5). ARGLU1 was found to occupy the *E2*, *E3,* and *E4* early gene promoters (Fig. 5) in *dl*309-infected HT-A cells, whereas the levels of ARGLU1 associated with these promoters were significantly reduced in *dl*1102-infected HT-A cells (Fig. 5). Interestingly, the levels of RPII at viral promoters were inversely correlated with viral gene expression, with higher levels observed in *dl*309-infected cells compared with *dl*1102-infected cells (Fig. 5). Additionally, E1A was consistently present at viral promoters with no significant difference observed between *dl*309 and *dl*1102 viruses.

### E1A binding to ARGLU1 inhibits DNA damage repair

**Fig 6:**
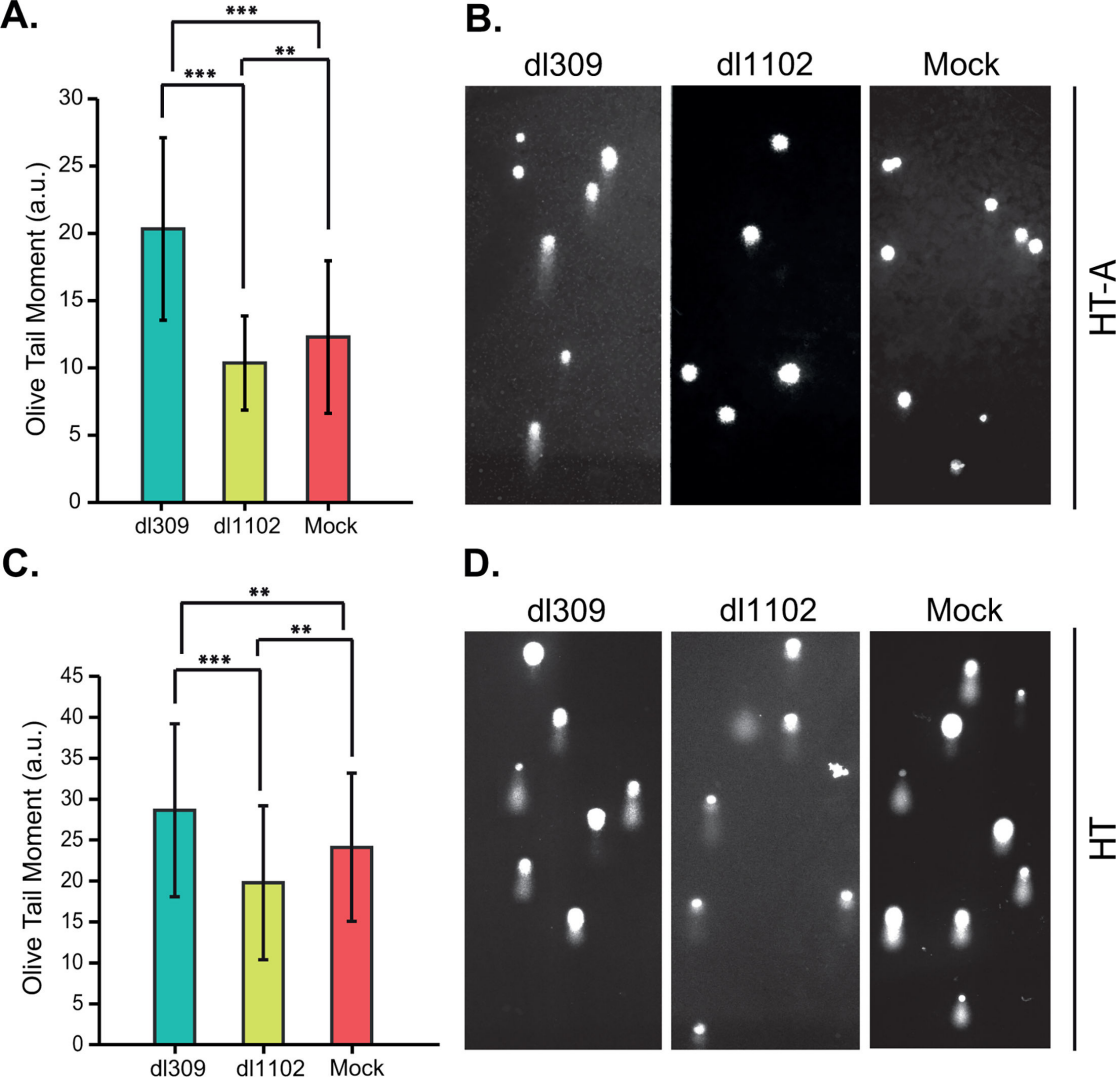
The ARGLU1–E1A interaction impairs cellular DNA damage repair. (**A**) HT-A cells were mock-infected or infected with HAdV5 *dl*309 or *dl*1102. At 24 h after infection, the cells were treated with bleomycin for 15 min, followed by the comet assay. DNA damage repair was assessed using the comet assay as described in Materials and Methods. Quantification represents comet Olive tail moments. Error bars indicate standard deviation (*n* = 100 comets); **P ≤ 0.01, ***P ≤ 0.001. a.u. = arbitrary units. (**B**) Representative images of comets from panel (**A**). (**C**) Same as (**A**) except the assay was performed in HT cells. Error bars indicate standard deviation (*n* = 100 comets); **P ≤ 0.01, ***P ≤ 0.001. a.u. = arbitrary units. (**D**) Representative images of comets from panel **C**.

We have previously demonstrated that ARGLU1 overexpression enhances DDR and increases cancer cell resistance to genotoxic drugs (3). One possibility for why E1A targets ARGLU1 is to interfere with DNA damage repair-related activities of ARGLU1, since DDR could have negative consequences for viral replication (19). We therefore investigated whether the binding of ARGLU1 by E1A during HAdV5 infection affects DDR. To explore this, bleomycin was used to induce DSBs in HT or HT-A cells thatb infected for 24 h with *dl*309 or *dl*1102, as we have done in the past (3). Comparison of DNA damage, performed by the comet assay as we did previously (3), shortly after removal of bleomycin showed that in *dl*309-infected cells, there were significantly higher levels of DNA damage compared with both mock and *dl*1102-infected cells (Fig. 6). The reduced repair in *dl*309-infected cells, but not *dl*1102-infected cells, suggests that the interaction of ARGLU1 with E1A potentially impairs the ability of ARGLU1 to contribute to DNA damage repair.

**Fig 7:**
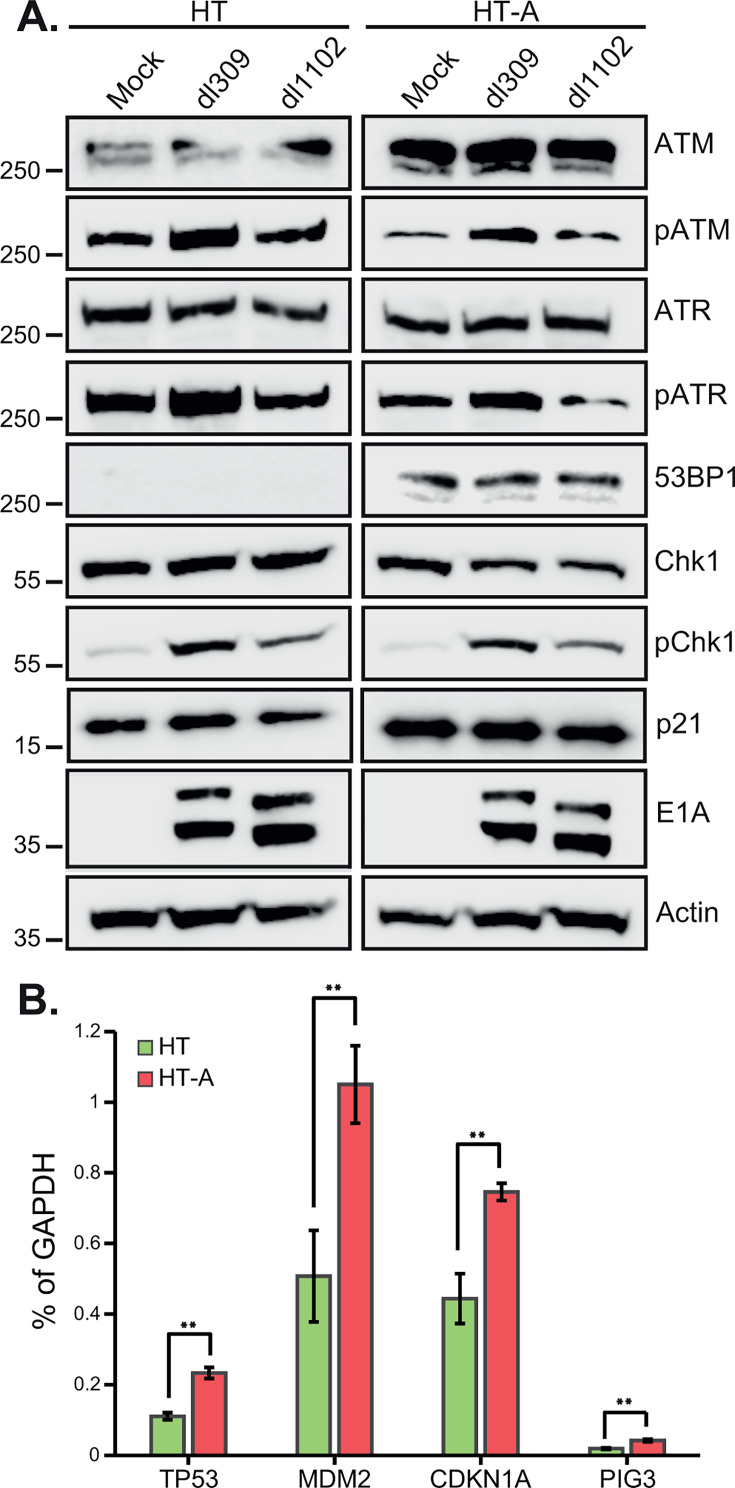
ARGLU1 overexpression enhances stress factor levels. (**A**) HT and HT-A cells were mock-infected or infected with HAdV5 *dl*309 or *dl*1102 at an MOI of 10. At 24 h after infection, the cells were lysed in NP-40 lysis buffer supplemented with phosphatase inhibitors as described in Materials and Methods; 20 µg of total protein was resolved on a 4%–12% NuPAGE BOLT gel and blotted for the indicated proteins. ACTIN was used as a loading control. (**B**) Cells treated in the same way as in (**A**) were subject to total RNA extraction using the TRIzol reagent, RNA was converted to cDNA using VILO Master Mix, quantified by qPCR, and normalized to GAPDH mRNA. The results are plotted as % of GAPDH mRNA. Error bars represent standard deviation (*n* = 3); **P ≤ 0.01.

DNA damage response is a complex signal transduction network that senses DNA damage via different members of the phosphatidylinositol 3-kinase-like protein kinase (PIKK) family, which includes ATM and ATR that become activated by autophosphorylation depending on the type of DNA damage (19, 31). Given our previous findings, we wanted to investigate whether ARGLU1 sequestering by E1A impairs DNA damage response by affecting activation, and therefore, phosphorylation, of ATM, ATR, and downstream Chk1, as well as downstream effectors such as p21 and 53BP1 (Fig. 7A). To explore this, we infected HT and HT-A cells with *dl*309 or *dl*1102 and assessed both total and phosphorylated protein levels of DNA damage response markers 24 h after infection. Levels of ATM and ATR were unchanged regardless of infection. However, levels of ATM were consistently higher in HT-A cells versus HT. Importantly, phosphorylation of ATM and ATR was enhanced in *dl*309-infected cells but largely unaffected by *dl*1102-infected cells compared with mock. The downstream target Chk1 was phosphorylated the most in *dl*309-infected cells, whereas Chk1 was moderately phosphorylated in *dl*1102-infected cells compared with mock. We also investigated other stress and DNA damage markers, specifically p21 and 53BP1. There was an overall higher level of the cell cycle inhibitor p21 in HT-A cells compared with HT. Levels of the DNA damage response factor 53BP1 were also higher in HT-A cells compared with HT cells, where the protein was undetectable. Infection had no significant impact on the overall levels of these proteins.

Finally, we investigated transcription of p53 target genes in HT and HT-A cells, as the elevated levels of p21 observed in HT-A cells suggested that p53-regulated genes may be activated (Fig. 7). Indeed, levels of p53-regulated gene transcripts for *TP53*, *MDM2*, *CDKN1A*, and *PIG3* were significantly upregulated in HT-A cells versus HT cells. This suggests that HT-A cells are more primed for stress response compared with the parental HT cells.

Overall, these results suggest that targeting of ARGLU1 by E1A reduces the DDR response in infected cells downstream of ATM/ATR activation and suggest that downstream stress effectors are upregulated in HT-A cells versus HT.

## DISCUSSION

In the present study, we have identified ARGLU1 as a direct interacting partner of HAdV5 E1A (Fig. 1). Binding of E1A to ARGLU1 recruits ARGLU1 to viral promoters, reducing viral gene expression likely via enhanced promoter-proximal pausing of RNA polymerase II, with subsequent reduction of viral protein levels. Consequently, we show that overexpression of ARGLU1 resulted in a significant reduction in virus growth without affecting viral genome replication (Fig. 2). Furthermore, we demonstrate that during infection, ARGLU1 sub-nuclear distribution is altered into distinct phenotypes not observed in uninfected cells (Fig. 4). Finally, our results suggest that targeting of ARGLU1 by E1A is important for inhibition of the DNA damage response pathway. Enhanced DNA damage repair may be responsible for reduced virus growth in ARGLU1-overexpressing HT-A cells.

Our previous studies showed that ARGLU1 promotes enhanced DNA damage repair through an increase in promoter proximal pausing of RPII after DNA damage has been induced (3). Enhanced DNA damage repair is undesirable for most viruses, as this can lead to viral genome replication defects and induce cell cycle arrest or apoptosis, leading to abortive infection (19). Therefore, viruses, including HAdV, aim to block this response during infection. Indeed, infection with HAdV *dl*309 led to reduced DNA damage repair following bleomycin treatment, but this was not the case with ARGLU1-binding-deficient E1A-expressing mutant *dl*1102 (Fig. 6). This suggests that E1A binding to ARGLU1 inhibits its activities that promote enhanced DNA damage repair. Curiously, binding of ARGLU1 by E1A leads to recruitment of ARGLU1 to viral promoters, reducing viral gene expression (Fig. 5), likely an undesirable outcome overall. However, this may be a consequence that ensures that DNA damage response is suppressed, and viral gene expression may not be the limiting factor in virus replication regardless. Importantly, recruitment of ARGLU1 to viral promoters correlates with enhanced presence of RPII on these promoters but overall reduced gene expression. This is not unexpected and is consistent with our earlier observations of ARGLU1 enhancing promoter proximal RPII pausing (3). RPII would not be expected to linger on viral promoters when it quickly transitions to elongating phase (7), but if it is paused there, it would be expected to have higher occupancy as we observed by ChIP (Fig. 5). We also aimed to determine at which point in the DNA damage response cascade ARGLU1 may participate, beyond promoting RPII pausing, by investigating activation of the DNA damage sensor kinases ATM and ATR and their downstream target Chk1. Although we observed no significant difference in the activation of ATM, ATR, and Chk1 upon infection, where infection with *dl*309 and *dl*1102 induced activating phosphorylation of these kinases, we did observe an unexpectedly higher level of ATM in HT-A cells compared with the parental cell line HT (Fig. 7A). This could increase the sensitivity of these cells to DNA damage and promote more efficient repair, but it does not provide a clear answer as to how ARGLU1 binding to E1A blocks DNA damage response beyond its impact on RPII pausing on viral promoters. It is possible that downstream signals may be affected, and this merits further investigation. Indeed, stress response effector molecules, such as p21 and 53BP1, were expressed at higher levels in HT-A cells compared with HT cells, suggesting that these cells are primed and ready to rapidly respond to stress or DNA damage.

Our previous characterization of the mutant *dl*1102 demonstrated that it is capable of replication to levels similar to those of wild-type-like *dl*309 (30). Interestingly, in that study, we observed that *dl*1102 expresses most of its viral genes to levels that are higher than *dl*309, which we have also observed here. At the time, it was unclear why that was, but our current study provides a plausible explanation. The loss of binding to ARGLU1 would result in less RPII pausing at the viral promoters, enhancing expression, as we observed here and in the past (3, 30). One possibility is that recruitment of ARGLU1 is not merely a serendipitous incident but is a consequence of natural selection that drives E1A to finely tune the viral transcriptional program. It is reasonable to assume that overactivation of certain genes could affect the host cell in undesirable ways and overall inhibit viral replication. By moderating transcription through ARGLU1 recruitment, the virus may optimize its gene expression for maximal viral replication in a living host. Curiously, *dl*1102 did not exhibit a growth deficiency or inability to drive S-phase in arrested IMR-90 cells (30). It is possible that higher viral gene expression may overcome any inability to block DNA damage response due to a deficiency in E1A binding to ARGLU1 in this mutant. Indeed, we have observed longer and more sustained expression of E1A in *dl*1102-infected cells (Fig. 3). Another possibility is that some degree of DNA damage response activation may be beneficial to the virus, and combined with other mechanisms, this may overcome any deleterious effects associated with loss of ARGLU1 binding to E1A. Indeed, activation of the FANC pathway has previously been shown to be beneficial to the viral replicative cycle (34). Clearly, the interaction of HAdV with the DNA damage response pathways is complex, and a deeper investigation will resolve these intriguing questions. Curiously, late protein levels were negatively impacted by ARGLU1 overexpression regardless of the virus infecting the cell (Fig. 3). Although somewhat unexpected, this demonstrates that late protein products are likely in vast excess and do not significantly impact viable viral yields. This may be a consequence of ARGLU1 impacting late transcript splicing, as its role in splicing regulation has been well established (2), and we have previously shown HAdV-dependence on splicing factor availability for specific splice variant production (35). This may lead to aberrant splicing that is not detectable by qPCR but leads to transcripts that are not properly translated into proteins.

In conclusion, the present study provides new insights into how HAdV re-tasks ARGLU1 to advance the viral replicative program. Our study identified ARGLU1 as a direct binding partner of HAdV5 E1A that is re-localized in the nucleus during viral infection, regardless of E1A binding, while providing further mechanistic insights into how ARGLU1 enhances cellular stress response. Moreover, our data further support the notion that we observed previously that ARGLU1 is a multifunctional regulator of viral transcription and cellular DNA damage response, with important implications for our understanding of virus–host interactions and suggests that ARGLU1 may be a valuable host restriction factor for antiviral therapy.

## Data Availability

All data used in the manuscript is contained in the figures.
